# A comparative study of microbial changes in dental plaque before and after single- and multiappointment treatments in patients with severe early childhood caries

**DOI:** 10.1186/s12903-024-04458-5

**Published:** 2024-06-15

**Authors:** Shi Ying Ma, Qing Nan Zhou, Shuang Cai, Yan Zhou, Xiao Yu Zhang, Xiao Yu Feng, Shu Diao, Jin Qiu Xi, Guo Xia Yu, Jia Jian Shang, Ning Yan Yang

**Affiliations:** 1 Department of Pediatric Dentistry, Beijing Stomatological Hospital & School of Stomatology, Capital Medical University, Beijing, 100050 China; 2 Department of Pediatric Dentistry, Stomatological Hospital affiliated Suzhou Vocational Health College, Suzhou, 215000 China; 3 Department of Stomatology, Beijing Children’s Hospital, Capital Medical University, National Center for Children’s Health, Beijing, 100045 China

**Keywords:** Severe early childhood caries, Dental treatment under general anaesthesia, Multiappointment treatment, 16S amplicon sequencing, Oral core flora

## Abstract

**Background:**

The status of dental caries is closely related to changes in the oral microbiome. In this study, we compared the diversity and structure of the dental plaque microbiome in children with severe early childhood caries (S-ECC) before and after general anaesthesia and outpatient treatment.

**Methods:**

Forty children aged 3 to 5 years with S-ECC who had completed whole-mouth dental treatment under general anaesthesia (C1) or in outpatient settings (C2) were selected, 20 in each group. The basic information and oral health status of the children were recorded, and the microbial community structure and diversity of dental plaque before treatment (C1, C2), the day after treatment(C2_0D), 7 days after treatment (C1_7D, C2_7D), 1 month after treatment (C1_1M, C2_1M), and 3 months after treatment (C1_3M, C2_3M) were analysed via 16 S rRNA high-throughput sequencing technology.

**Results:**

(1) The alpha diversity test showed that the flora richness in the multiappointment group was significantly greater at posttreatment than at pretreatment (*P* < 0.05), and the remaining alpha diversity index did not significantly differ between the 2 groups (*P* > 0.05). The beta diversity analysis revealed that the flora structures of the C1_7D group and the C2_3M group were significantly different from those of the other time points within the respective groups (*P* < 0.05). (2) The core flora existed in both the pre- and posttreatment groups, and the proportion of their flora abundance could be altered depending on the caries status of the children in both groups. *Leptotrichia* abundance was significantly (*P* < 0.05) lower at 7 days posttreatment in both the single- and multiappointment groups. *Corynebacterium* and *Corynebacterium_matruchotii* were significantly more abundant in the C1_1M and C1_3M groups than in the C1 and C1_7D groups (*P* < 0.05). *Streptococcus, Haemophilus* and *Haemophilus_parainfluenzae* were significantly more abundant in the C1_7D group than in the other groups (*P* < 0.05).

**Conclusion:**

A single session of treatment under general anaesthesia can cause dramatic changes in the microbial community structure and composition within 7 days after treatment, whereas treatment over multiple appointments may cause slow changes in oral flora diversity.

## Background

Severe early childhood caries (S-ECC) is a serious disorder that affects children younger than 6 years old, and its occurrence is related to genetic, environmental (family environment, social environment), and microbiological factors [1]. Clinically, nonpharmacological behavioural management methods are typically used several times to treat children with S-ECC with the aim of gradually improving their chewing function and reducing the possibility of caries. As parents pay more attention to children’s oral health, children are being diagnosed at increasingly younger ages; if children cannot cooperate with conventional clinical operations and there are too many decayed teeth, they can choose to receive a single dental treatment under general anaesthesia. The clinical efficacy of single- and multiappointment treatments differs, and caries tend to recur in the long term after treatment; studies have shown that the recurrence rate can reach 59% at 1 year after successful treatment for caries [2, 3].

The presence of oral microorganisms is a prerequisite and is the main aetiological risk factor for the development of caries. Caries derive from the joint action of various microorganisms in the oral cavity and are a pathological manifestation of the disruption of the oral microecological balance [4]. Under normal conditions, various microorganisms cohabitate, compete, and antagonize dental plaque and maintain a dynamically balanced microecological system in terms of population, number and function. Changes in local environmental factors can shift the ratio of flora and metabolites within plaque biofilms; this shift can cause dysbiosis of the dental plaque biofilm ecology, the evolution of normal bacteria into conditional pathogens, and the development of dental disease [5].

Changes in the diversity and composition of floral genera are closely related to caries status, and there are differences in the microbial community and composition of plaques between caries and caries-free children [6]. However, most studies on caries in younger children is cross-sectional methods. Those studies have mainly focused on revealing the differences in oral microbiome between different caries status populations [7, 8]. Then, knowledge of the oral microbiome shift with caries development process is limited. Only a few of longitudinal studies is reported. Xu et al. [9] reported a longitudinal study of a 1-year follow-up of 3-year-old caries-free children and found drastic microbial shifts occur during caries development, as well as in the sub-clinical state. Another cohort study investigated the changes of the oral ecosystem before and after using fluoride varnish in S-ECC children at 5 different time points, revealed that health-associated *Bacteroides* and *Uncultured_bacterium_f_Enterobacteriaceae* were enriched in the saliva samples [10]. Therefore, the shift of oral microbiom may be used for predicting caries-related activity among children to determine their caries risk.

Under general anaesthesia, the treatment of multiple teeth in the mouth often occurs during a single appointment, and the intraoral microbial environment may change greatly within a short time. Conventional outpatient treatment may gradually change the structure and diversity of the flora, and these two different forms of change are important for studying the recurrence process of caries and the long-term efficacy of caries treatment. Therefore, the aims of this study are as follows: to investigate the clinical efficacy of two different caries management methods through a follow-up study of children with S-ECC who were treated once under general anaesthesia and those who were treated across multiple appointments; to investigate the differences in oral supragingival plaque microorganisms before and after caries treatment via 16 S rRNA gene sequencing technology; and to screen for biomarkers of caries and the caries-free state to provide guidance for the treatment and prevention and monitoring of caries.

## Materials and methods

### Patient recruitment and sampling

This study included forty children aged 3–5 years with S-ECC who received full-mouth dental treatment under general anaesthesia during one visit or outpatient treatments from September 2021 to January 2023 at the Department of Pediatric Dentistry, Beijing Stomatological Hospital, Capital Medical University. The inclusion criteria were as follows: (1) good general health (ASA class I), no history of systemic diseases and genetic diseases, no history of systemic medication, no maxillofacial malformation; (2) age 3–5 years, a complete row of deciduous teeth, with ≤ 1 previous filling; and (3) 8 ≤ dmft ≤ 16, with both primary anterior and molar teeth caries. The exclusion criteria for patients were as follows: (1) parents or guardians requested to treat only partially decayed teeth for various reasons and (2) had systemic application of antibiotics or fluoride treatment within 2 weeks before treatment. This study was approved by the Medical Ethics Committee of Beijing Stomatological Hospital, Capital Medical University (CMUSH-IRB-KJ-PJ-2020-17).

### Dental examination

Two examiners (physicians who had at least 1 year of clinical experience in paediatric dentistry and who had been uniformly trained; the standard concordance test kappa values were > 0.8) performed the oral health examination under the light of the dental chair using forceps, a dental mirror, and a probe. The caries damage of the children was examined and recorded according to the WHO caries diagnostic criteria [11]. The caries loss and filling indices of the children were counted (decayed, missing, and filled tooth index, dmft). The diagnostic criteria were as follows: a caries was defined as a lesion with a soft bottom in the sulcus or smooth surface of the tooth and potential damage to the enamel or a softening of the sulcus wall. White spots on the enamel, uneven areas of staining, stained sulci that can be inserted by a probe but whose bottom is not soft, and hard depressions on the enamel caused by moderate to severe fluorosis were not diagnosed as caries.

### Clinical sample collection methods and steps

The children involved in the study were screened and informed in advance to not brush their teeth on the morning of the sampling day. Caries state and plaque samples were collected from 8:00–10:00 a.m. The children rinsed their mouths with water, a sterile cotton ball, and a saliva suction device to isolate the wetness before sampling, referring to the standard sampling procedure of the Human Microbiome Project (HMP) (http://www.hmpdacc.org/HMP Clinical Protocol. pdf, Jul 29, 2010). The supragingival plaque was collected with a sterile spatula from the intact enamel of all the tooth surfaces in sterilized 1.5 mL Eppendorf tubes containing 0.2 mL Tris-HCl buffer (pH = 8.0), and all samples mixed with blood and other residues were discarded. The samples were temporarily stored at −20 °C for 2 h and subsequently transferred to the laboratory for freezing and storage at −80 °C.

### Dental treatment and posttreatment follow-up

The children in both groups were treated by experienced paediatric dentists according to the standardized treatment plan. The whole treatment course was controlled within 1 month in separate treatment sessions. The children in both groups were reviewed at 7 days, 1 month, and 3 months after treatment. During the review, the physician performed an oral examination to collect supragingival plaque from the children and treated the children with recurrent caries. In addition, the parents of the children were educated on oral hygiene, including brushing and flossing methods, abstaining from bad eating habits, and being informed of the importance of regular oral health checkups. The clinically collected samples were divided into two groups according to the treatment modality and follow-up time points: (1) single-appointment group: pretreatment (C1), 7 days posttreatment (C1_7D), 1 month posttreatment (C1_1M), and 3 months posttreatment (C1_3M); and (2) multiappointment group: pretreatment (C2), same day posttreatment (C2_0D), 7 days posttreatment (C2_7D), 1 month posttreatment (C2_1M), and 3 months posttreatment (C2_3M).

### DNA extraction and sequencing

Approximately 60 uL of supragingival plaque samples were collected and total DNA from the bacterial population was extracted using KF kit B (Magen Biotechnology, Guangzhou, China), and the DNA concentration was detected using the Qubit™ dsDNA BR Assay Kit (Invitrogen, CA, USA) to screen for samples with a DNA concentration of > 20 ng/µL. The DNA that passed the test was used to amplify the 16 S rRNA V3-V4 region using polymerase chain reaction (PCR) with primers 338 F (5’-ACTCCTACGGGGAGGCAGCAG-3’), 806R (5’-GGACTACHVGGGTWTCTAAT-3’). The amplification products were purified with magnetic beads and constructed into libraries using the Miseq Reagent Kit v3 (Illumina, CA, USA), and then bipartite sequencing was performed on the Illumina MiSeq PE300 platform (BGI, Shenzhen, China) to produce 2 × 300 bp paired-end reads.

### Bioinformatic and statistical analysis

The data were filtered, and then clustered into operational taxonomic units (OTUs) using USEARCH (v 7.0.1090), and classified according to Silva (v 138.1) as reference databases Annotation. Based on the OTU and annotation results, sample species complexity analysis was performed, species differences between groups were determined, and association analysis and model prediction were performed.

With SPSS 22.0 and R language 4.2.2 statistical software, the normality test was performed using the S–W test, and the nonparametric test was applied to analyse the measurement data that did not conform to the normal distribution (such as the age of the child at the first visit, the caries index before treatment, and the abundance of key difference species at each classification level). The measurement data that were normally distributed (such as the alpha diversity index of the flora between different subgroups) were analysed via one-way analysis of variance (ANOVA) for comparative analysis, and the chi-square test was used for comparative analysis of count data (e.g., children’s compliance rate and children’s and parents’ basic information), with a test efficacy of α = 0.05. β-Diversity level analysis was performed for differences in community structure among different subgroups, and analysis of similarities (ANOSIM) and permutation were applied. Permutational multivariate analysis of variance (PERMANOVA) was used for nonparametric tests.

## Results

### Basic information of the children and follow-up results

The demographic data of the patients in the single- and multiappointment groups in the study are shown in Table 1. A total of 40 children with S-ECC were included in this study, 20 in the single-appointment group and 20 in the multiappointment treatment group, and all children in the single-appointment group completed the follow-up at each time point 3 months after surgery. While in the multiappointment treatment group, 4 individuals were lost for follow-ups at 7 days and 1 month posttreatment, and 6 were lost at 3 months. There were no significant differences between the two groups in terms of sex, age at first visit, or clinical characteristics (i.e., dmfs, parents’ education level, the child’s main caregiver, the reason for the child’s first dental visit and posttreatment caries rate); however, the number of decayed, missing, or filled teeth was greater in the C1 group than in the C2 group. However, caries recurrence were observed in both single- and multiappointment groups as early as 3 months after treatment.

**Table 1:** Pretreatment baseline and posttreatment caries incidence between the single- and multiappointment treatment groups [M(Q25, Q75)/n]

Group	First visit age (months)	Gender (M/F)	dmft	dmfs	Posttreatment review (caries free/caries recurrence)		
					7 days	1 month	3 months
Single-appointment group (*n* = 20)	48 (41, 55)	6/14	13.5 (12.0, 15.0)	29.0 (21.0, 45.0)	20/0	20/0	13/7
Multiappointment group (*n* = 20)	54 (45, 59)	12/8	10.0 (8.0, 13.0)	23.5 (16.5, 34.8)	16/0	16/0	10/4
P value	0.104	0.112	**0.005**	0.101	1.000	1.000	0.693

dmft: number of decayed, missing, or filled teeth; dmfs: number of decayed, missing, or filled tooth surfaces

M: mean value; Q25: 25% quartile; Q75:75% quartile

### Analysis of the structure and diversity of the bacterial flora

#### OTU statistics

A total of 4,010,393 high-quality DNA sequences were obtained in the single-appointment group by the Illumina HiSeq sequencing platform, and the effective sequence lengths were concentrated between 452 and 461 bp (mean 457 bp). A total of 505 OTUs were identified, with 336 OTUs shared among the four groups(C1, C1_7D, C1_1M, and C1_3M). A total of 4,109,327 high-quality DNA sequences were obtained in the multiappointment group, and the effective sequence lengths ranged from 415 to 424 bp, with a mean of 420 bp. A total of 892 OTUs were identified in the five groups (C2, C2_0D, C2_7D, C2_1M, and C2_3M). After taxonomic assignment, we identified a total of 16 phyla, 23 classes, 59 orders, 103 families, 250 genera, and 312 species in the single- and multiappointment treatment groups.

### *Microbial diversity level*

**Fig. 1:**
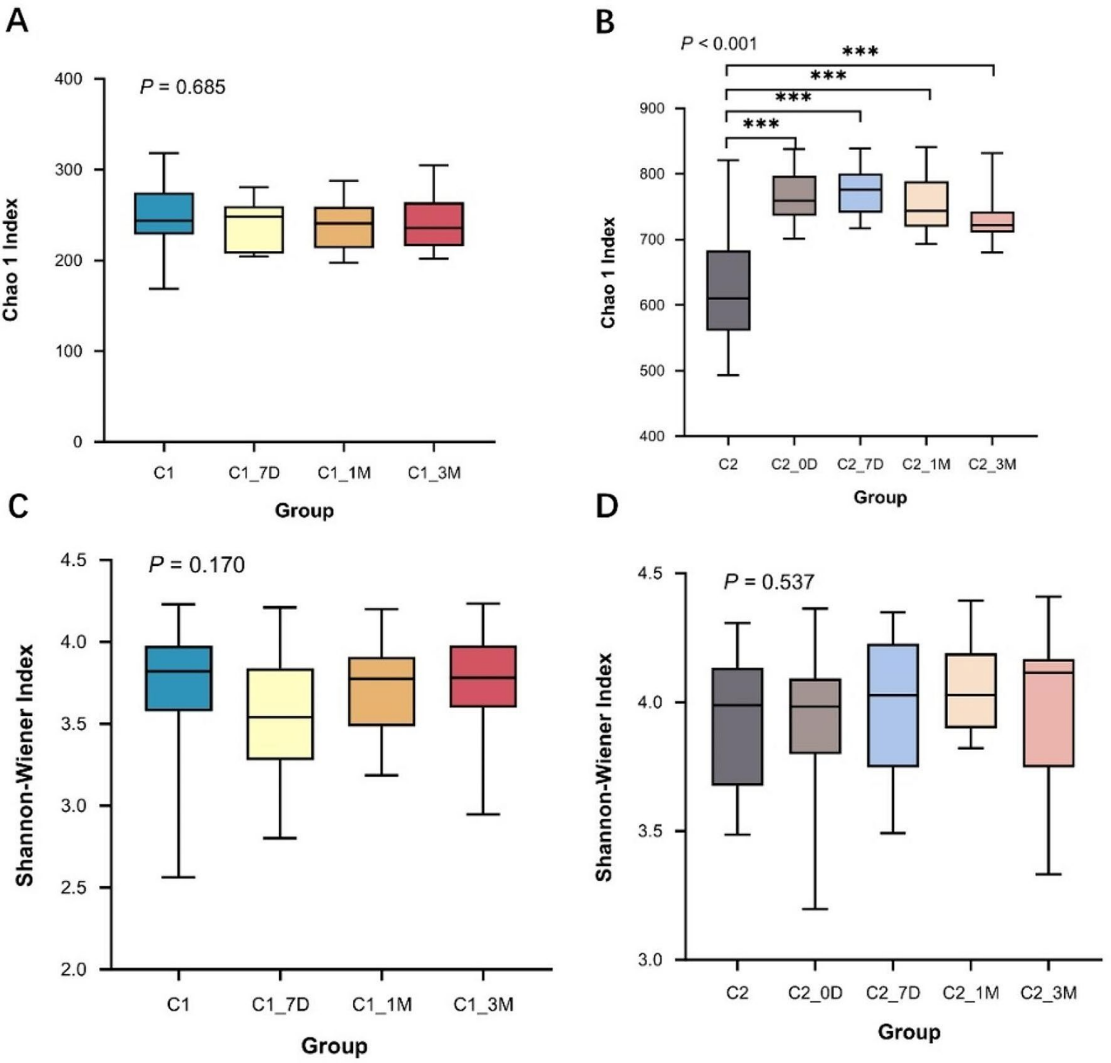
Comparison of alpha diversity indices between groups before and after treatment. The figure includes the chao1 index (**A**, **B**) and Shannon index (**C**, **D**). (**A**, **C**) Single-appointment group; (**B**, **D**) Multiappointment group

Based on the OTU results, the estimated species richness index (Chao1) and species diversity index (Shannon index) were calculated, and the results showed that the Chao1 index in the multiappointment group started to decrease after reaching the maximum at 7 days posttreatment; however, the values at all posttreatment time points were greater than those before treatment and were statistically significant (*P* < 0.001), indicating that the posttreatment microbial community richness in the multiappointment group was greater than that before treatment, whereas the difference of the Shannon index between pre- and posttreatment was not significant. And, there was no significant difference between the Chao1 index and Shannnon index before and after treatment in the single-appointment group (*P*>0.05), indicating that there was no obvious change in species richness and evenness before and after treatment (Fig. 1).

**Fig. 2:**
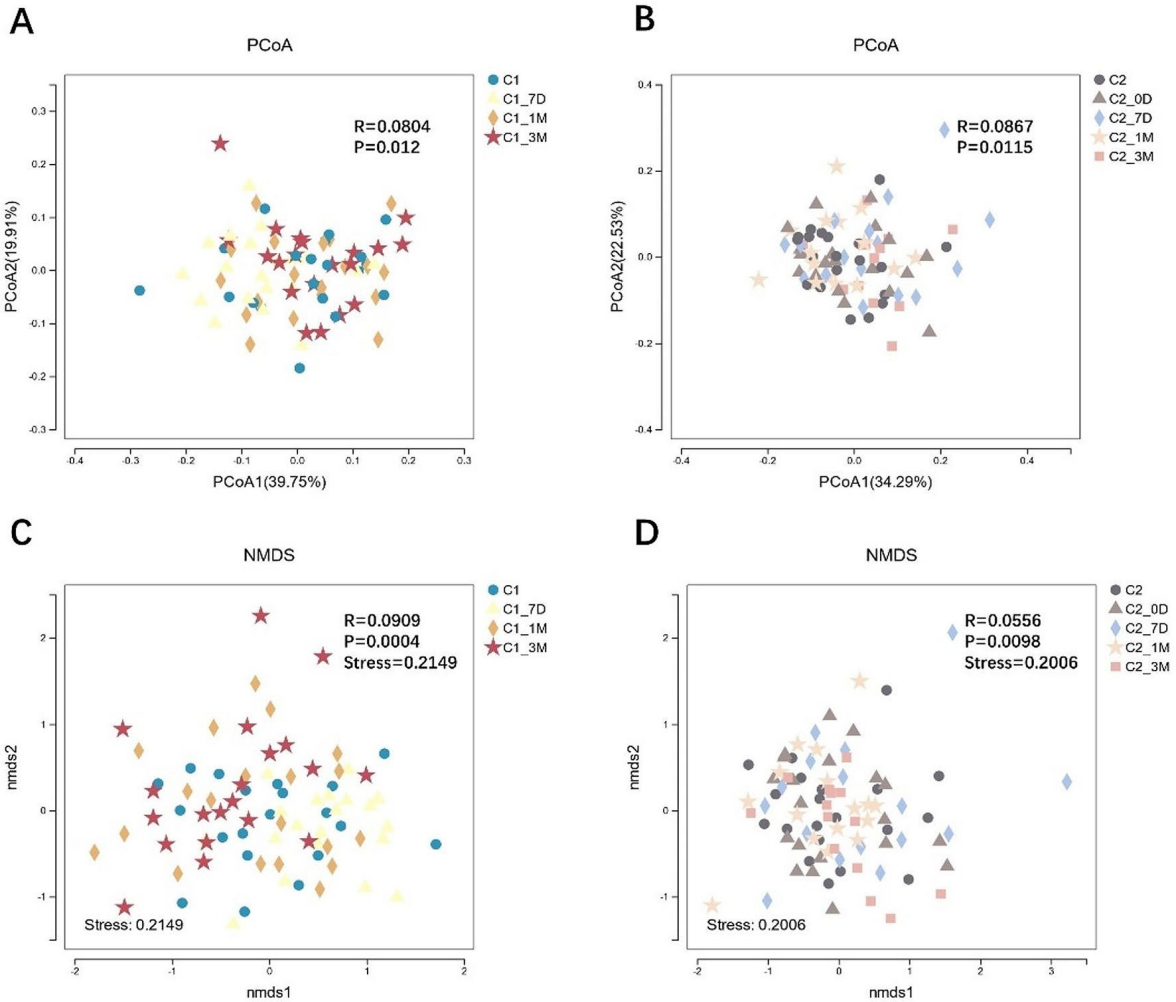
Comparison of beta diversity indices between groups before and after treatment. The figure includes main axis analysis results (**A**, **B**) and NMDS analysis results (**C**, **D**). (**A**, **C**) Single-appointment group; (**B**, **D**) Multiappointment group

PCoA based on a weighted UniFrac distance matrix and NMDS analysis based on the Bray–Meier algorithm can be used to identify similarities or differences in the data. The results of the study showed that the single- and multiappointment treatment groups had similar distances and similar compositions of plaque samples. The differences between the groups were greater than the differences within groups (|R| > 0), and the differences in the microbial community species composition between the groups were significant (*P* < 0.05). Similarly, the difference in community species structure between C1_7D and all the other time points was significant in the single-appointment group (*P* < 0.05), as well as the difference (*P* < 0.05) between C2_3M and all the other time points in the multiappointment group (Table 2; Fig. 2).

**Table 2:** Statistical results of two-way comparisons of PCoA and NMDS within the single- and multiappointment groups

Group	PERMANOVA		ANOSIM	
	R2	*P*	*R*	*P*
C1/C1_7D	0.0751	**0.0172**	0.1388	**0.0023**
C1/C1_1M	0.0230	0.4695	0.0073	0.3522
C1/C1_3M	0.0393	0.1503	0.0267	0.1529
C1_7D/C1_1M	0.0787	**0.0117**	0.1348	**0.0019**
C1_7D/C1_3M	0.1380	**0.0001**	0.2762	**0.0001**
C1_1M/C1_3M	0.0104	0.8922	-0.0371	0.9260
C2/C2_0D	0.0280	0.3319	0.0615	**0.0463**
C2/C2_7D	0.0635	0.0543	0.1101	**0.0130**
C2/C2_1M	0.0264	0.4666	0.0108	0.3410
C2/C2_3M	0.0825	**0.0127**	0.0811	0.0664
C2_0D/C2_7D	0.0493	0.1207	0.0656	0.0685
C2_0D/C2_1M	0.6333	**0.0201**	0.0093	0.3589
C2_0D/C2_3M	0.0849	**0.0123**	0.0787	0.0667
C2_7D/C2_1M	0.0690	0.0650	0.0253	0.2071
C2_7D/C2_3M	0.0525	0.1676	0.0729	0.0546
C2_1M/C2_3M	0.1237	**0.0015**	0.0424	0.1411

### *Microbial community composition analysis*

**Fig. 3:**
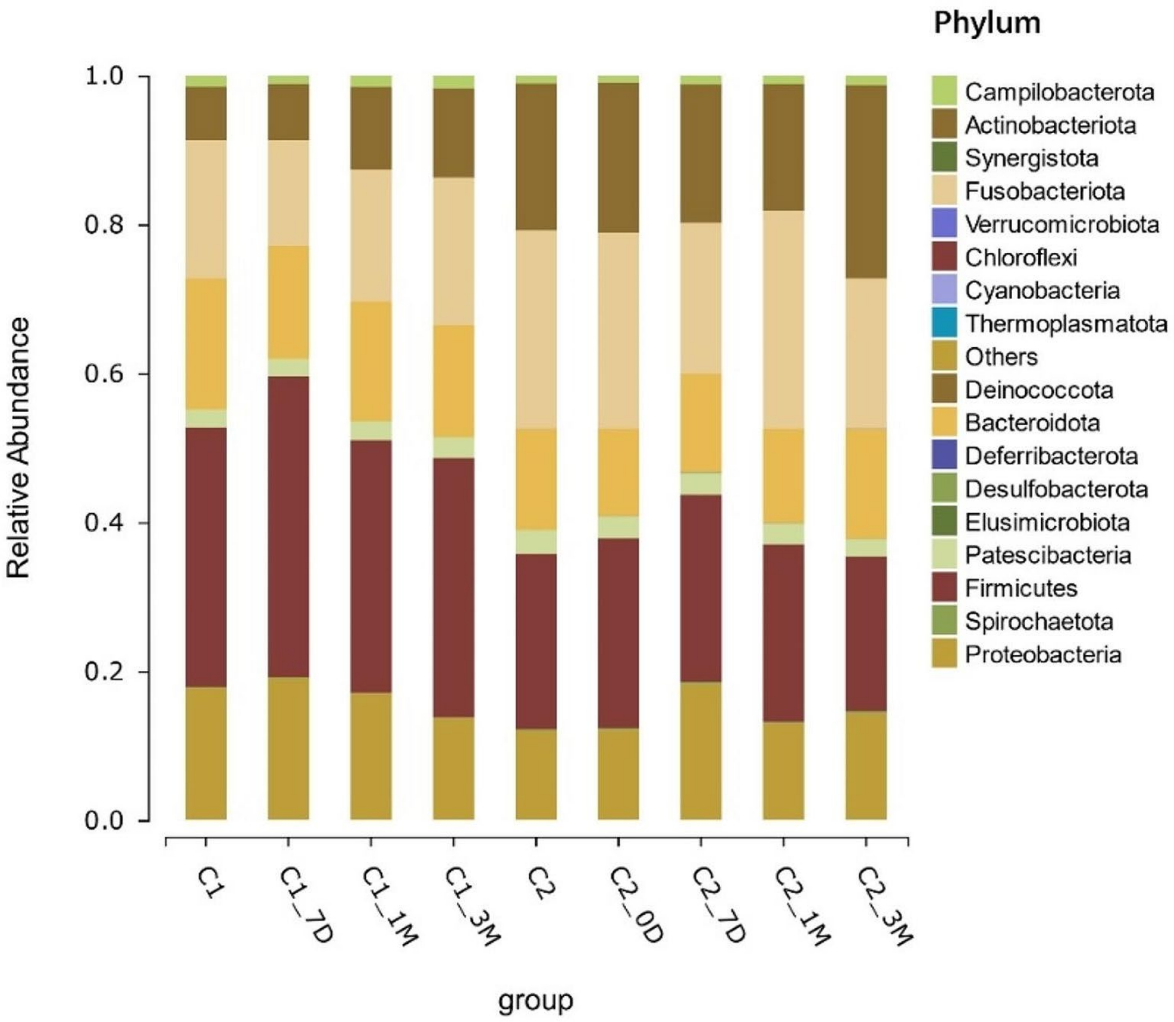
Relative distribution of microorganisms under the phylum level in single- and multiappointment groups.

For every taxonomic tier, ranked by abundance, the bacteria with the higher abundance were selected to show the average relative abundance of each group and tested for differences. In this study, we found that the phyla *Firmicutes*, *Fusobacteria*, *Proteobacteria*, *Bacteroidota*, and *Actinobacteriota* were highly abundant in both the single- and multiappointment groups. The five major phyla, amounting to more than 95%, were the main dominant phyla, followed by *Campilobacterota* and *Patescibacteria* (Fig. 3).

**Fig. 4:**
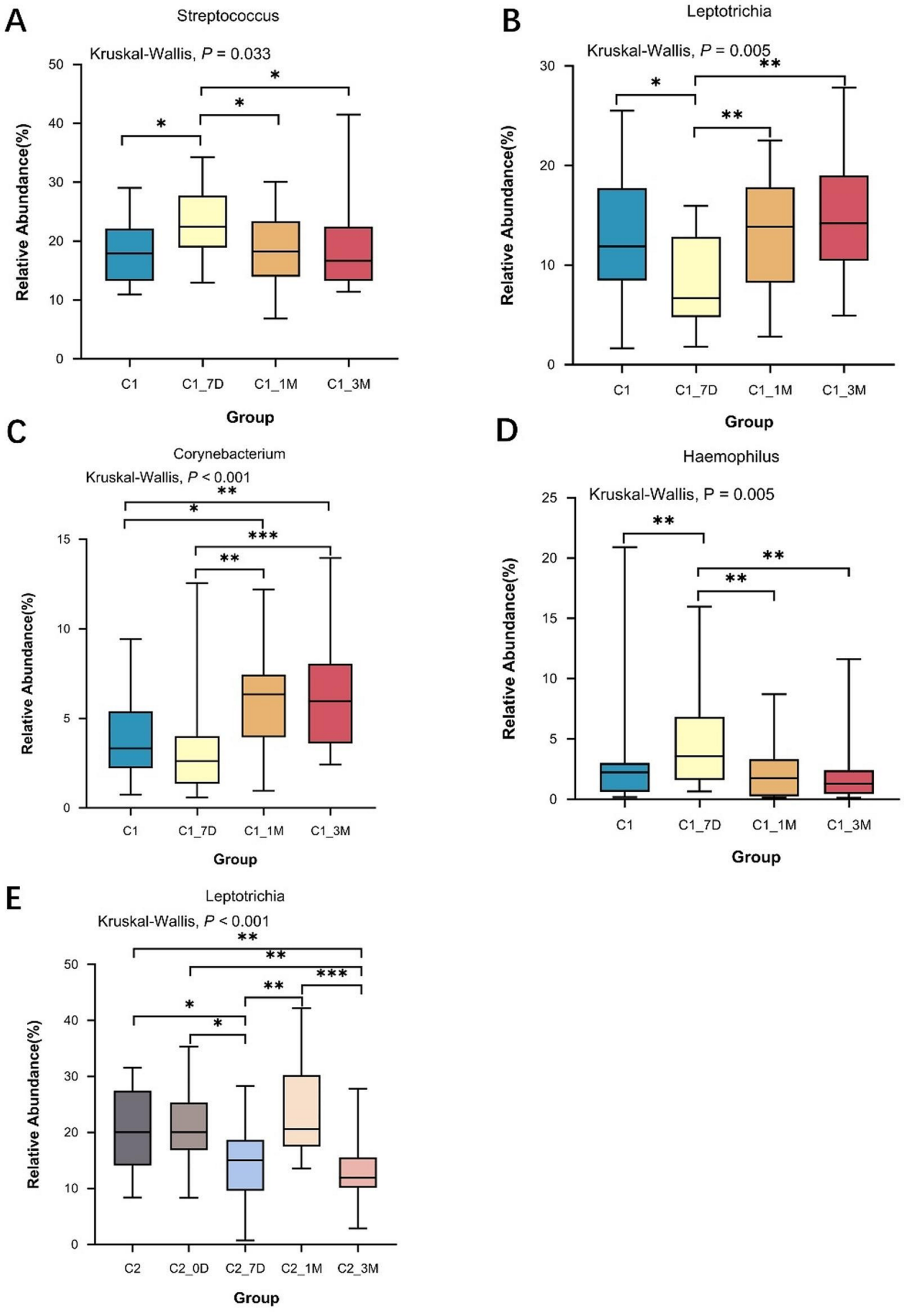
Distribution of key difference species at the genus level for single- and multiappointment groups. (* indicates *P* < 0.05; ** indicates *P* < 0.01; *** indicates *P* < 0.001) (**A**, **B**, **C**, **D**) Single-appointment group; (**E**) Multiappointment group

At the genus level, *Streptococcus*, *Leptotrichia*, *Neisseria*, *Veillonella*, *Capnocytophaga*, *Prevotella*, *Fusobacterium, Actinomyces, Selenomonas, Haemophilus* and *Corynebacterium* were the dominant genera, accounting for more than 90% of all genera. The distributions of *Streptococcus*, *Leptotrichia*, *Corynebacterium*, and *Haemophilus* were significantly different between the children before and after the single-appointment group (*P* < 0.05). The relative abundance of *Corynebacterium* was significantly greater at 1 and 3 months after treatment than at pretreatment and 7 days posttreatment (*P* < 0.05). Similarly, the relative abundances of both *Haemophilus* and *Streptococcus* reached their highest levels at 7 days after treatment (*P* < 0.05) and returned to their pretreatment levels at 1 and 3 months after treatment, while the relative abundance of *Leptotrichia* reached its lowest level at 7 days after treatment (*P* < 0.05) and increased to pretreatment level at 1 and 3 months after treatment (Fig. 4A–D). In the multiappointment group, the distribution of *Leptotrichia* was significantly different between the children before and after treatment, with the lowest relative abundance occurring at 7 days after treatment and a rapid increase occurring at 1 month after treatment (*P* < 0.01) (Fig. 4E).

**Fig. 5:**
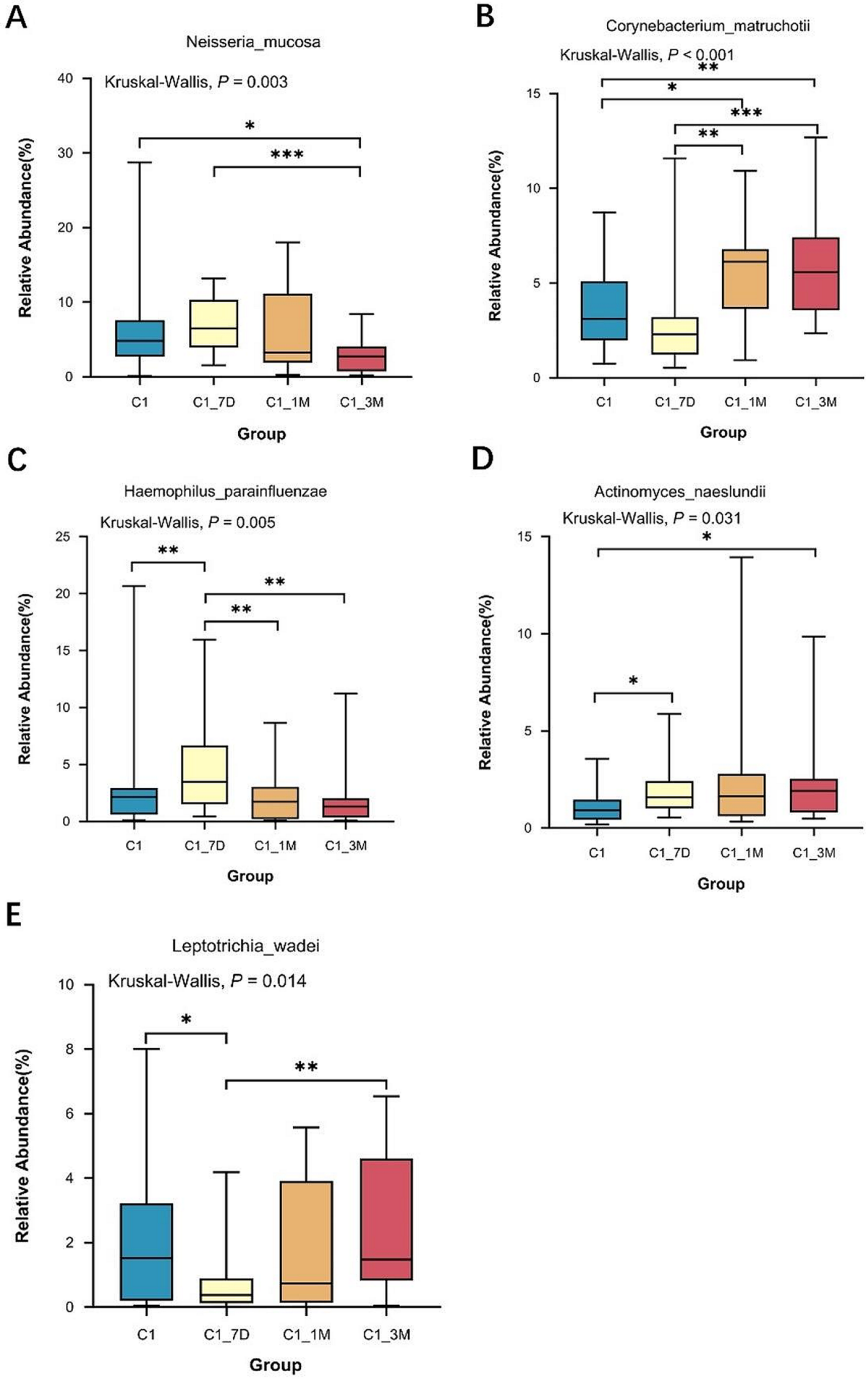
Distribution of key difference species at the species level for single-appointment group

The top 10 species were *Veillonella_parvula*, *Neisseria_mucosa*, *Fusobacterium_nucleatum*, *Corynebacterium_matruchotii*, *Haemophilus_parainfluenzae*, *Leptotrichia_sp*, *Actinomyces_naeslundii*, *Capnocytophaga_granulosa*, *Leptotrichia_buccalis*, and *Leptotrichia_wadei*, accounting for 30-50%, but their proportions differed between the two groups of children. At the species level, a significant difference (*P* < 0.05) was found in the distributions of *Neisseria_mucosa*, *Corynebacterium_matruchotii, Haemophilus_parainfluenzae*, *Actinomyces_naeslundii*, and *Leptotrichia_wadei* before and after treatment in children in the single-appointment group. The relative abundance of *Leptotrichia_wadei* reached a minimum at 7 days posttreatment (*P* < 0.05) and increased to pretreatment level at 1 month after treatment, whereas, the relative abundance levels of *Corynebacterium_matruchotii* haven’t changed at 7 days posttreatment and increased at 1 and 3 months after treatment (*P* < 0.01). Similarly, the relative abundance levels of *Haemophilus_parainfluenzae* reached highest at 7 days posttreatment and gradually decreased at 1 and 3 months posttreatment (*P* < 0.01), whereas the relative abundance of *Neisseria_mucosa* was significantly lower at 3 months posttreatment (*P* < 0.01), and furthermore, the relative abundance of *Actinomyces_naeslundii* gradually increased during the posttreatment period (*P* < 0.05) (Fig. 5). However, at the species level, there was no significant difference in the relative distribution of the dominant species before and after treatment in the multiappointment group (*P* > 0.05).

## Discussion

S-ECC children usually have the option of completing their dental treatment under general anaesthesia and in outpatient sessions. Our study focused on the dynamic change of microbes after treatment, and we collected pre- and posttreatment supragingival plaque samples for analysis. The main flora and microbiological diversity of the caries site are significantly different from the normal site and the aggregated plaque on the surfaces of the filled restorations [12–14]. However, most of the tooth surfaces in the posttreatment group were caries-free, so we collected caries-free supragingival plaque for observation to ensure comparability before and after treatment. In addition, on the day of general anaesthesia, cleaning during treatment removes the plaque. For a period of short time after treatment, the child is unable to eat normally, resulting in insufficient time for plaque formation. Therefore we set the first review point at 1 week (C1_7D). In contrast, in the multiappointment group, the treatment process is slow and may last up to 1 month, making it necessary to collect supragingival plaque on the day after treatment (C2_0D).

Dental caries in children have high recurrence even after receiving perfect treatment, and the incidence of new caries can reach 53% 1–2 years after full-mouth caries treatment [15]. Liu et al. [16] retrospectively analysed 844 children aged 0–12 years who underwent systematic treatment with general anaesthesia or restraint local anaesthesia and reported that the compliance rate was greater in children treated with caries under general anaesthesia than in those treated under restraint local anaesthesia. Our study also revealed that caries recurrence occurred 3 months after treatment in both groups and that children in the multiappointment group had lower compliance with follow-up examinations. However, very little is known about the shift of microbiome after dental treatment. This was the first study to investigate the changes in the microbiological profile of dental plaque in children with S-ECC before and after the completion of full-mouth dental treatment in one or multiple sessions.

The alpha diversity results showed that the microbial evenness did not change before and after treatment in either the single or multiappointment groups, while the posttreatment microbial community Chao1 index of children in the multiappointment group was greater than that before treatment. The richness of the flora increased in the caries-free state after treatment, which might be related to the fact that surgical intervention removing key caries-causing bacteria, resulting in the growth of other microorganisms. The beta diversity results showed that the microbiological structure of the flora changed within a short period of time at 7 days posttreatment in the single-appointment group, whereas the microbiological structure of the flora changed within 3 months posttreatment in the multiple-treatment group. These suggest that caries treatment may primarily alter the relative abundance and structure of species in the existing microbial community rather than eliminating existing taxa or introducing new taxa into the microbial community. Jiang et al. [17] found that the abundances of only certain groups of bacteria differed between caries and caries-free children through the analysis of salivary flora, and there was no significant difference in overall diversity, similar to the results of the present study. A study by Shi [18] also revealed that microbial diversity was related to different microecological spatial sites and was not significantly influenced by different caries levels. Therefore, the caries severity and sampling site may have affected the microbial alpha diversity analysis, and additional in-depth studies are still needed.

By comparing and annotating the OTU representative sequences with the Silva database, we found that the phylum levels were mainly concentrated in the five major phyla *Firmicutes, Fusobacteria, Proteobacteria, Bacteroidota*, and *Actinobacteriota* in both groups before and after treatment, reaching more than 95%; this was followed by *Campilobacterota* and *Patescibacteria*, and proportional changes in the abundance of different phyla were observed at different time points after treatment. At the phylum level, the composition of oral microorganisms was essentially stable across caries states, which is consistent with the results of previous studies [19–21].

At the genus level, 20 genera, including *Streptococcus, Leptotrichia, Neisseria*, and *Veillonella* were found to be predominant before and after treatment in both the single- and multiappointment groups, and the relative abundance of *Leptotrichia*, which belongs to the *Fusobacteriota*, decreased at 7 days after treatment in both groups of children. Similarly, Zhang et al. [22] studied the difference in oral flora between caries and caries-free groups of children aged 2–6 years by 16 S rRNA sequence analysis and found that it was significantly greater in the caries group than in the caries-free group, which was similar to our study, suggesting that *Leptotrichia* may be potential caries-causing bacteria in the oral cavity. In the single-appointment group, the relative abundance of *Haemophilus* was significantly greater at 7 days posttreatment than pretreatment, whereas the relative abundance of *Corynebacterium* was significantly greater at 1 and 3 months posttreatment than pretreatment. Studies by Li et al. [23] and Schoilew et al. [24] respectively found that the enrichment of *Haemophilus* and *Corynebacterium* may be a marker of oral health status, which confirms our findings.

The top 10 species before and after treatment in both groups of children were *Veillonella_parvula, Neisseria_mucosa, Fusobacterium_nucleatum, Corynebacterium_matruchotii, Haemophilus_parainfluenzae, Leptotrichia_sp, Actinomyces_naeslundii*, *Capnocytophaga_granulosa, Leptotrichia_wadei* and *Leptotrichia_buccalis*, but their percentages of abundance differed between the two groups. For example, the relative abundance of *Haemophilus_parainfluenzae* increased significantly at 7 days posttreatment, and that of *Corynebacterium_matruchotii* increased significantly at 1 and 3 months posttreatment. Mark et al. [25] reported that *Corynebacterium_matruchotii* could be present in large numbers on the surface of healthy enamel and in supragingival plaque, suggesting that it may act as the dominant group in the caries-free state. The abundance of *Actinomyces_naeslundii* also tends to increase gradually after treatment, while previous studies have linked it to the development of caries in children [26, 27]. In addition, *Neisseria_mucosa* was significantly lower at 3 months posttreatment and *Leptotrichia_wadei* was significantly lower at 7 days posttreatment in our study. A recent study demonstrated that *Corynebacterium_matruchotii*, *Actinomyces_naeslundii*, and *Haemophilus_influenzae* significantly increased, and *Streptococcus_mutans*, three species from *Leptotrichia*, and *Neisseria_bacilliformis* significantly decreased 1 month after general anaesthesia treatment in S-ECC children, which is quite similar to the results of our study [28]. *Streptococcus_mutans* is considered to be the main cariogenic bacteria [29, 30], but it was neither the dominant species nor showed significant changes before and after treatment in the present study. Ratson et al. [31] also found the abundance of *Streptococcus_mutans* and *Streptococcus_sobrinus* in children with S-ECC at 3 months after comprehensive dental treatment decreased slightly but not statistically significant, and they concluded that the majority of these children retained this cariogenic microbiota after the operation. Therefore, further studies may be needed to confirmed the role of *Streptococcus_mutans* in the dynamics of caries before and after treatment.

In conclusion, the distribution of the dominant bacterial species in the oral cavity of children can change within a short period of time after completing dental treatment in a single session, but this change is not continuous or stable. However, there are some limitations in this study, such as some samples were lost to visit resulting in less comprehensive study results. In addition, 16s rRNA high-throughput sequencing technology was in our study olnly identified the bacteria in the sample. In recent years, fungi (such as *Candida albicans* and *Candida dubliniensis*) and viruses (such as *Cytomegalovirus* and *Epstein-Barr virus*) have also been reported to be linked to dental caries [32–36]. Therefore, future studies should be strengthened in follow-up management, and focus more on the correlation between other microorganisms and dental caries on the other.

## Conclusion

In summary, the flora structure of children with S-ECC is complex. A single treatment under general anaesthesia can cause dramatic changes in the oral microbial community structure (including taxonomic composition and relative abundance), and the composition of the oral core microbial community (including *Leptotrichia*, *Corynebacterium*, *Corynebacterium_matruchotii*, *Streptococcus* and *Haemophilus*) within 7 days after treatment. Multiple sessions of outpatient treatment may cause slow changes in oral flora diversity (mainly accompanying changes in *Leptotrichia*). A comparative study of the different patterns of changes in oral flora before and after single- and multiappointment treatment will help us to reveal the long-term clinical efficacy of these two treatments for caries management at the aetiological level and to screen for caries-related biomarkers.

## Data Availability

Raw sequencing data during the current study are available in the National Center for Biotechnology Information (NCBI) Sequence Read Archive (SRA) database (accession number PRJNA1095515). Patient data are available from the corresponding author upon reasonable request.
